# Importance of additional risk factors in postoperative atrial fibrillation risk analysis methods

**DOI:** 10.1590/1806-9282.20231319

**Published:** 2024-02-26

**Authors:** Mesut Engin, Nazlı Cansu Gürsoy, Ahmet Burak Tatlı

**Affiliations:** 1 University of Health Sciences Bursa Yuksek Ihtisas Training and Research Hospital, Department of Cardiovascular Surgery - Bursa, Turkey.

Dear Editor,

We have read the article entitled “Blood group as a novel predictor of postoperative atrial fibrillation after off-pump coronary artery bypass grafting” by Donmez and Muduroglu^1^ with great interest. First, we congratulate the authors for their valuable contributions. However, we would like to discuss the risk factors of postoperative atrial fibrillation (PoAF) after coronary artery bypass graft (CABG) surgery.

Various studies on blood groups have been conducted in the literature. Due to the close relationship between blood group antigens and inflammation, the relationship between blood type and cardiovascular disease has been investigated. Non-O-type blood groups have been shown to be associated with cardiovascular diseases and susceptibility to thrombosis^2^. Atrial fibrillation is also known to be associated with inflammation^3^. A study conducted by Jang et al. showed a significant relationship between AB blood groups and thromboembolic events in patients with nonvalvular atrial fibrillation^4^. In their current study, Liu et al. demonstrated a significant relationship between AB blood groups and recurrence in patients who underwent catheter ablation due to atrial fibrillation^5^. However, in this current article, the authors revealed for the first time in the literature a relationship between PoAF and blood groups after open heart surgery^1^. This is a valuable information, and some points need to be clarified in order to guide future studies. We think that known risk factors should be clearly defined and analyzed in studies where a new risk factor is shown as an independent predictor.

PoAF is an important problem after CABG operations, and factors such as age, chronic obstructive pulmonary disease, hypertension, and heart failure are important preoperative risk factors^6^. Apart from these factors, factors such as the patient’s medical treatments in the perioperative period, use of blood products, and use of positive inotropic agents also affect the PoAF status^7^ ^,^ ^8^. To what do the authors attribute the similar hypertension rates (50.8 vs. 49%) between the two groups in their study? Were the patients’ antihypertensive drug use rates similar in the preoperative period? For which diseases did the authors use the definition of “chronic pulmonary disease” from their study? How was “heart failure” defined in the study? The rate of patients with heart failure was higher in the non-PoAF group, although it was not statistically significant (29.6 vs. 24.6%, p=0.225). Why do the authors think this situation occurs? How did the authors define the variable “chronic renal dysfunction” in their study? Was the value of blood creatinine above 2 mg/dL? Should patients receive hemodialysis? The authors also found the rate of need for positive inotropic agents to be similar between groups (21.8 vs. 31.1%, p=0.056). How was the “inotrope requirement” variable defined in the study? Finally, the authors found that the number of distal anastomoses was similar between the groups (3.64±1.19 vs. 3.72±1.17, p=0.528). Did the authors calculate the SYNTAX score I value in their study group, which is an important factor to determine the complexity of coronary artery disease^6^ and a risk factor for PoAF?
